# Advances in Understanding How Heavy Metal Pollution Triggers Gastric Cancer

**DOI:** 10.1155/2016/7825432

**Published:** 2016-10-10

**Authors:** Wenzhen Yuan, Ning Yang, Xiangkai Li

**Affiliations:** ^1^Department of Oncology Surgery, First Hospital of Lanzhou University, Lanzhou, Gansu 730000, China; ^2^The life Science School of Lanzhou University, Lanzhou, Gansu 730000, China

## Abstract

With the development of industrialization and urbanization, heavy metals contamination has become a major environmental problem. Numerous investigations have revealed an association between heavy metal exposure and the incidence and mortality of gastric cancer. The mechanisms of heavy metals (lead, cadmium, mercury, chromium, and arsenic) contamination leading to gastric cancer are concluded in this review. There are four main potential mechanisms: (1) Heavy metals disrupt the gastric mucosal barrier by decreasing mucosal thickness, mucus content, and basal acid output, thereby affecting the function of E-cadherin and inducing reactive oxygen species (ROS) damage. (2) Heavy metals directly or indirectly induce ROS generation and cause gastric mucosal and DNA lesions, which subsequently alter gene regulation, signal transduction, and cell growth, ultimately leading to carcinogenesis. Exposure to heavy metals also enhances gastric cancer cell invasion and metastasis. (3) Heavy metals inhibit DNA damage repair or cause inefficient lesion repair. (4) Heavy metals may induce other gene abnormalities. In addition, heavy metals can induce the expression of proinflammatory chemokine interleukin-8 (IL-8) and microRNAs, which promotes tumorigenesis. The present review is an effort to underline the human health problem caused by heavy metal with recent development in order to garner a broader perspective.

## 1. Introduction

With the development of industrialization and urbanization, heavy metal contamination is a major environmental problem that affects organisms metabolism in ecosystems due to its high toxicity, prevalence, and persistence existence [1, 2]. In generally, metals with a density >5 g/cm^3^ are considered heavy metals. Among them, lead (Pb), cadmium (Cd), mercury (Hg), chromium (Cr), and arsenic (As) broadly exist in environment and are considered to be the primary toxic heavy metals to human health [1–3].

**Figure 1:**
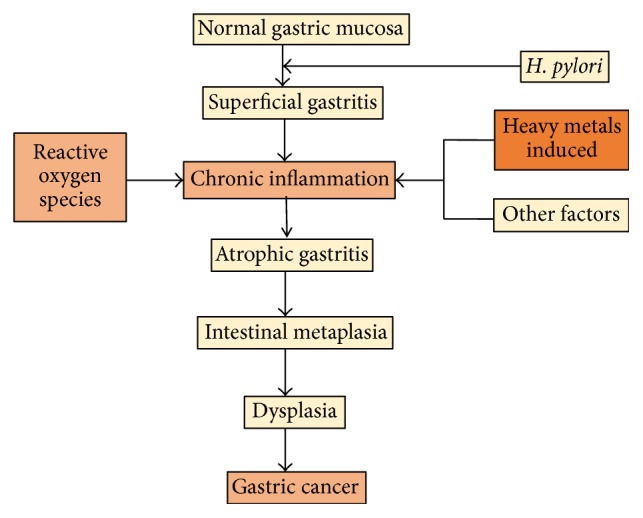
The key steps in the development of gastric cancer and the factors that influence this development [5, 6].

Gastric cancer is the second most common cause of cancer-related death worldwide and the fourth most common malignancy among cancers, especially in Asia. In 2008, there were approximately one million new gastric cancer patients. Worldwide, there were 738,000 deaths from gastric cancer; almost 47% of these gastric cancer cases occurred in China [4]. The majority of gastric cancers are adenocarcinomas and they are generally classified as either intestinal- or diffuse-type. The development of gastric cancer is a complex and multifactorial process, involving numerous lesions such as superficial gastritis, chronic inflammation, atrophic gastritis, intestinal metaplasia, dysplasia, and carcinoma (Figure 1) [5]. The etiology of gastric cancer is multifactorial and the predisposing factors include high levels of nitrates, high salt intake, smoking,* Helicobacter pylori* infection, and a familial genetic component, which accounts for a small percentage of patients [5, 6].

Previous research has indicated that heavy metal exposure damages the development of the nervous, hematological, and cardiovascular systems and increases the risk of numerous cancers including kidney, lung, liver, skin, and gastric cancer [1–3]. Some metals, such as Cr, Pb, As, Cd, and Hg, have been classified as certain or probable carcinogens by the International Agency for Research on Cancer [1, 3]. Many investigators stated that heavy metal exposure increased the incidence and mortality of gastric cancer (Table 1). The results of a meta-analysis indicated that Cr^6+^ exposure increased the risk of gastric cancer (relative risk [RR] = 1.41, 95% confidence interval [CI] 1.18–1.69) [3]. Topsoil concentrations of Cr correlated with mortality in women with upper gastrointestinal (GI) tract cancer. Long-term exposure to low levels of As and Cr in topsoil could be a potential risk factor for developing cancer [7]. Between 2005 and 2010, soil levels of As significantly correlated with the mortality rates of gastric, colon, kidney, nasopharyngeal, and lung cancer in Suzhou, China [8]. A recent ecological study demonstrated a positive correlation between As levels in soil and gastric cancer (*P* = 0.412); an increase of 1 ppm As concentration in the soil was associated with an 11.1% increase in the gastric cancer mortality rate (RR = 1.111, 95% CI 1.061–1.165) [8]. Long-term exposure to Cd and Pb enhanced the mortality risk of several cancers, including lung, esophageal, and gastric cancer in a region surrounding a multimetal sulphide mine [9]. Compared with healthy people, gastric cancer patients in Tabriz, northwestern Iran, had higher urine concentrations of Cd. A multivariate regression model revealed a significant association between urinary Cd concentration and gastric cancer risk [10]. The highest death rate associated with gastric cancer was reported in the cities of Divandareh, Bijar, and Saghez. Concentrations of Pb, As, and antimony (Sb) in these cities were higher than those reported in others cites [11]. Statistical analysis has shown that the majority of liver, bladder, and stomach cancers in villages located in the Simav Plain in Turkey were associated with high As concentrations in the drinking water [12]. Meanwhile, mortality statistics collected from this region for years 1995 to 2005 showed that the rate of GI cancers was higher than the Turkish average [13]. Heavy metal pollution of soil, fruits, and vegetables has led to a high rate of GI cancers in Turkey [14]. A population-based case-control study indicated a correlation between inorganic Pb exposure and gastric cancer (odds ratio [OR] = 3.0, 95% Cl 1.2–7.3; and OR = 2.0; 95% CI 1.1–3.8, resp.) among male patients with gastric cancer [15]. Mortality analysis on wastewater exposure between 2007 and 2011 showed that malignant neoplasms and gastric cancer mortality and disease odds were higher in wastewater areas than in clean water areas (OR = 1.7, 95% CI 1.3–2.2,* P* < 0.01 and OR = 1.8, 95% CI 1.2–2.7,* P* < 0.01, resp.), in Shijiazhuang, Hebei, China [16]. There was a statistically significant association between Pb topsoil levels and incidence of human gastric cancer, while Hg topsoil levels were associated with the incidence of human liver cancer [17]. Meanwhile, other reports have revealed that changes in hair Hg levels may reflect the transition of normal gastric mucosal to superficial gastritis and atrophic gastritis, or even cancer [18]. Chronic low-dose exposure to heavy metals may play an important role in the process of tumorigenesis, and high concentrations are not required to induce tumorigenesis [17].

In summary, the incidence and mortality rates of gastric cancer are increasing in areas that are exposed to heavy metals. In this review, we present a comprehensive overview of the possible mechanisms by which heavy metals exposure, including Pb, Cd, Hg, Cr, and As, may contribute to gastric cancer development.

## 2. Mechanisms of Heavy Metal Carcinogenicity in the Stomach

### 2.1. Disruption of the Gastric Mucosal Barrier

**Figure 2:**
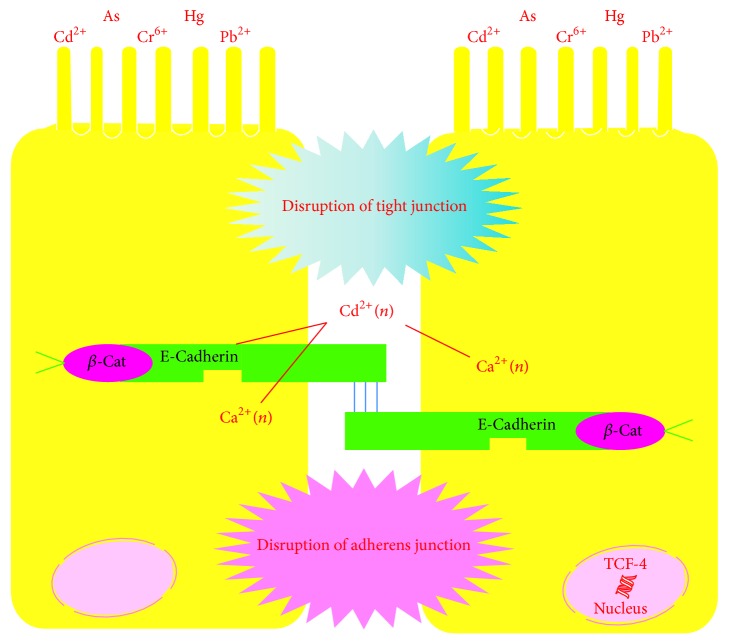
Disruption of the gastric mucosal barrier by heavy metals [19–21].

Heavy metals such as Cd^2+^ can displace Ca^2+^, thereby affecting the function of E-cadherin and disrupting cell junctions (Figure 2) [19]. In this manner, the gastric mucosal barrier is disrupted due to decreased mucus thickness, mucous content, and basal acid output and increased lipid peroxidation products [19–21]. One study reported that Cd exposure led to intestinal inflammation and tissue damage. It has been recently reported that chronic Cd exposure resulted in the formation of dysplastic lesions in gastric glandular epithelium but that it did not damage the esophageal and intestinal epithelium [26]. When rats were exposed to 15 ppm CdCl_2_ for 30 days, mean blood and mucosa Cd levels significantly increased (*P* < 0.01 and *P* < 0.001, resp.), while mucus thickness, mucous content (*P* < 0.01), and basal acid output (*P* < 0.01) significantly decreased. Cd-induced changes in the gastric mucosa can disrupt the gastric barrier and increase the risk of ulceration [20]. Cd also disrupts tight cell junctions by affecting the function of E-cadherin and subsequently triggering *β*-catenin-mediated activation of oncogenes in epithelial cells. Cd-induced disruption of E-cadherin affects cell-cell junctions and may represent a key step in both the cancer initiating and tumor promoting properties of Cd [19]. There is a significantly positive correlation between the presence of Cd-induced lipid peroxidation products and mucosal Cd (*r* = 0.664, *P* < 0.01). Compared with unexposed animals, mucous content and prostaglandin levels in the mucosa, which are ingredients of the gastric mucosal barrier, significantly decreased in Cd-exposed animals. Cd-induced lipid peroxidation impairs the gastric mucosa, disrupting the mucosal barrier and increasing the vulnerability of the gastric mucosa [21].

**Figure 3:**
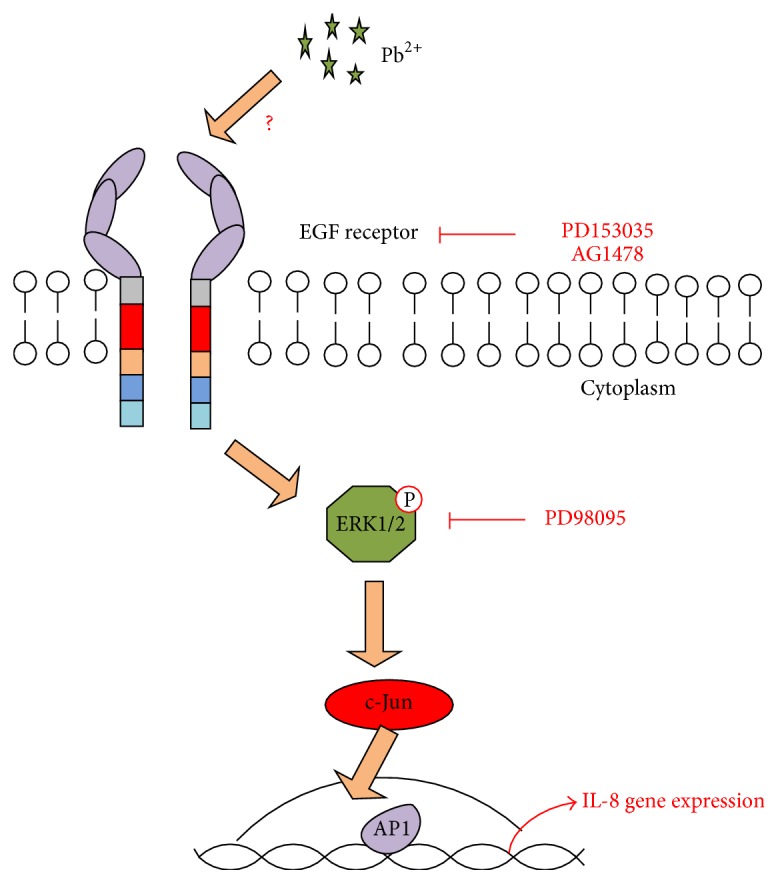
Schematic representation of the signaling pathways involved in Pb^2+^-mediated IL-8 expression [22].

Recently, it has been reported that Pb^2+^ can promote tumorigenesis, by inducing interleukin-8 (IL-8) expression in gastric cancer AGS cells. IL-8, a proinflammatory chemokine, promotes tumor metastasis and angiogenesis. Activator protein-1 (AP-1) and extracellular signal-regulated kinase play critical roles in signal transduction in Pb^2+^-induced IL-8 gene activation in human gastric cancer (Figure 3) [22]. Pb^2+^ activates epidermal growth factor receptor (EGFR) and induces phosphorylation of downstream extracellular signal-regulated kinase (ERK)-1/2, thereby activating AP-1. Overexpression of c-Jun increases activation of the gastrin promoter [37].

Cr^6+^ is reduced to Cr^3+^ in the acidic environment of the stomach and acts as a detoxifying agent. However, Cr^6+^ may not be completely reduced to Cr^3+^; approximately 10–20% of Cr^6+^ escapes gastric detoxification due to kinetic competition from Cr^3+^ absorption and gastric emptying and enters the systemic circulation [40]. Cr^6+^ does not react with DNA* in vitro* or in isolated nuclei. However, in the presence of cellular reductants, Cr^6+^, inside the cell, induces a wide variety of DNA lesions, including Cr-DNA adducts, DNA-protein cross-links, DNA-DNA cross-links, and oxidative damage [23, 41]. It has been shown by comet assay that Cr^6+^ can induce DNA lesions in peripheral blood lymphocytes and human gastric mucosa cells partly due to direct contact between Cr^6+^ and the stomach mucosa, at a concentration of 500 mM (*P* < 0.001) [42, 43].

### 2.2. Reactive Oxygen Species

**Figure 4:**
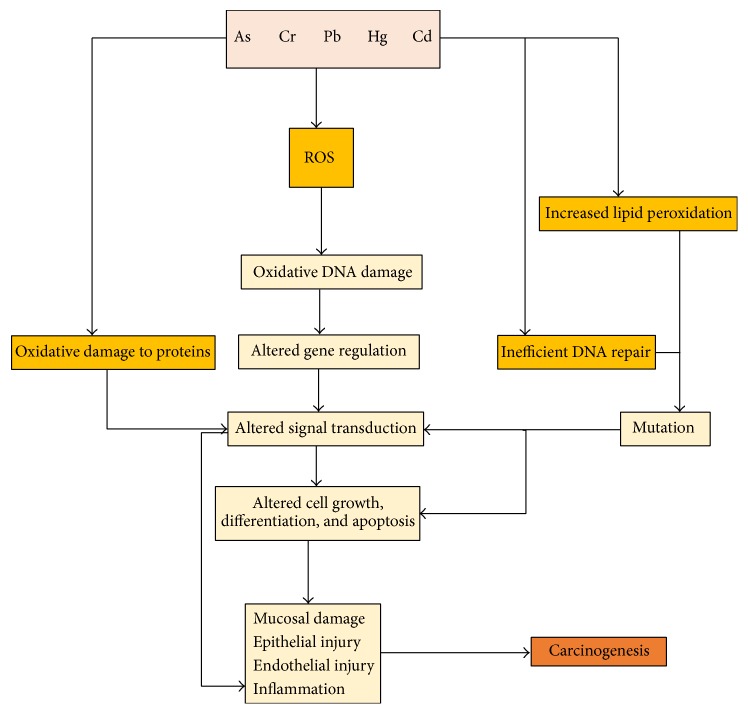
Mechanisms by which heavy metals induce gastric cancer via the generation of ROS [21, 23, 24].

Reactive oxygen species (ROS) are byproducts of normal cellular metabolism. Low and moderate amounts of ROS are beneficial because they kill invading pathogens and induce wound healing and tissue repair [24]. In contrast, at high concentrations, ROS can mediate damage to cell structures, lipids and membranes, proteins, and nucleic acids [23]. The cumulative generation of ROS, either endogenously or exogenously, induces a cellular redox imbalance in various cancers [23]. There are numerous external triggers that induce oxidative stress, thereby directly or indirectly affecting the GI tract [23]. Heavy metals, including Pb, Cd, Hg, Cr, and As, are common exogenous sources of ROS and can directly or indirectly induce ROS generation [23]. Exogenous ROS generation causes gastric mucosal damage, including epithelial and endothelial injury and inflammation (Figure 4). Various GI diseases including peptic ulcers, GI cancers, and inflammatory bowel disease may be caused in part by ROS. When the accumulation of ROS, either exogenously or endogenously, surpasses the patient's antioxidant levels, gene regulation is altered. This, in turn, induces changes in signal transduction and cell growth, ultimately leading to carcinogenesis (Figure 4) [24]. Furthermore, the formation of ROS disrupts metal ion homeostasis, overcoming the body's antioxidant protection. Subsequently, lipid peroxidation and protein modification processes are increased, causing numerous diseases such as chronic inflammation, diabetes, gastric cancer, and other cancers [23].

As-induced generation of free radicals can cause cell damage and death through activation of oxidative stress-sensitive signaling pathways [23]. There is definitive evidence that chronic As exposure leads to genomic instability, such as DNA lesions, ineffective DNA repair, telomere dysfunction, and abnormal mitosis, via the generation of oxidative stress [31]. Cd can replace iron and copper in various cytoplasmic and membrane proteins, thus increasing the amount of unbound, free, or chelated copper and iron ions which then participate in oxidative stress via Fenton reactions. Experimental evidence has indicated that, even at low concentrations, Cd can induce mutations by inducing DNA damage and inhibiting DNA lesion repair [27].

Positive dose-response associations have also been reported between Cr levels in erythrocytes and Olive tail moment, tail length, and percentage of tail DNA in electroplating workers in Hangzhou, China [44]. Mercuric chloride, a major source of mercury, provoked DNA damage in peripheral blood leucocytes in rat, which is visualized by comet assay [45]. Chromosomal aberrations can be caused by different types of amalgam, and high copper-amalgam is associated with a higher frequency of chromosomal aberrations than conventional amalgam [34]. By measuring* in vivo* urinary 8-hydroxy-2-deoxyguanosine levels, one study determined that Hg-induced DNA damage is linked with the production of free radicals [46]. Low levels of methyl mercury (100 *μ*g/day) can lead to significant DNA damage induction and decreased glutathione peroxidase (GSH-Px) activity in rats [35].

Even low levels of Pb can induce DNA double helix damage in mice, as shown through comet and DNA-protein cross-link assays. This is because Pb can bind to DNA by electrostatic forces and combine with purines and pyrimidine to access the DNA minor grooves [38]. Other reasons include changes in the cellular redox state, GSH, or downregulation of PKCa that indirectly induce genotoxicity. The length of time of Pb exposure significantly correlated with DNA damage in a study of 45 workers exposed to Pb (*r* = 0.690, *P* < 0.01) [47]. Pb and Cd provoke a* mutagenic* effect in cell, which increased clastogenic/aneugenic effect in peripheral lymphocytes [48]. Even if the levels of Pb are safe, coexposure with Cd and cobalt induces single strand DNA breaks and ineffective DNA repair [49].

Furthermore, research has indicated that catalase is involved in antioxidant defense mechanisms and may be related to certain tumor processes. It prevents accumulation of excessive levels of ROS at the cellular and tissue level. Exposure to nickel (Ni), Pb, Hg, and Cd alters catalase activity by generating high ROS levels in gastric cancer [28].

### 2.3. Ineffective DNA Repair

Cd can also act as an antagonist to alter the Cd/Zn ratio, which in turn induces high error rates and ineffective DNA repair. In the tumor suppressor protein p53, Zn can be replaced by Cd, thereby inducing DNA damage in the p53 gene, affecting its binding activity and repair-related processes [50]. Cd acts as a mitogen, stimulating cell proliferation, inhibiting apoptosis and DNA repair, and promoting cancer in number of tissues. It also causes tissue damage, notably in the kidney, by inducing cell death [29]. At the cellular level, Cd affects cellular proliferation and differentiation and causes apoptosis. It does this by inducing damage to DNA, cell membranes, and proteins, by inhibiting different types of DNA repair, and by inducing apoptosis in Cd-containing mammalian cells. Hartwig et al. proposed a novel mechanism of metal-related carcinogenicity characterized by inactivation of polymerases by metal compounds at nanomolar concentrations [50]. Meanwhile, Cd is capable of inhibiting DNA methylation leading to enhance clonal expansion of damaged and mutated cells, thus accelerating cancer development [27]. Pb acetate induces nicks in chromosomal DNA, as determined by a nick translation assay in cells [39]. In combination with ultraviolet radiation, Pb can also inhibit DNA repair [51].

The p53 tumor suppressor protein is decreased in the stomach of rats treated with Cr^6+^, as determined by western blotting. Numerous studies have shown a connection between c-Myc deregulation and gastric cancer. Galectin is a key factor in malignancy, including liver, stomach, and colon cancer. The expression of c-Myc (*P* < 0.05) and galectin-1 (*P* < 0.05) was increased in the stomach of rats treated with Cr^6+^ [52]. Cd can accelerate cancer development by activating protooncogenes and genes involved in cell proliferation* (c-myc, c-jun)* and by inhibiting DNA methylation, which increases clonal expansion of damaged and mutated cells, thus accelerating cancer development [27].

### 2.4. Gene Abnormalities

Genes involved in cancer-related pathways are more often influenced by epigenetic alterations than by genetic alterations in gastric cancer [53]. Long-term exposure to Cr may enhance histone deacetylation due to chronic cross-linking of inhibitory complexes. This phenomenon leads to histone methylation in specific positions involved in gene repression and silencing and to subsequent DNA hypermethylation, which then evolves into a complete and efficient state of gene silencing [40]. Such changes could cause epigenetic and structural alterations leading to changes in gene transcription. Ultimately, if these changes occur within critical cell cycle regulatory genes, this leads to Cr-mediated transformation and carcinogenesis, due to, for example, indirect silencing of the tumor suppressor gene* MLH1* [54]. Carcinogenic metals (Ni, As, and Cr) can evoke posttranslational histone modifications and modulate histone-modifying enzymes including iron- and 2-oxoglutarate-dependent dioxygenase family enzymes, DNA repair enzymes ABH3 and ABH2, and histone methyltransferases, which may affect the epigenome [32]. Furthermore, As can induce proapoptotic gene silencing or mutation, which leads to the survival of damaged cells [31]. Mercuric dichloride induced a significant increase in DNA migration in human salivary gland tissue cells and lymphocytes in carcinogenesis, as determined by the comet assay. Mercuric dichloride at 10 and 12 mg/kg body weight (BW) significantly induced several kinds of chromosomal aberrations, including chromatid and chromosomal breaks, clumps and damaged cells, and comets in rats [55]. Hg may also induce immune system dysfunction by inducing apoptosis in immune cells [36]. Banfalvi et al. reported that Pb can cause chromatin changes in K562 cells [56]. When clone B3 from EJ30 bladder cell carcinoma cell line was exposed to Pb, there was a significant induction in telomere-induced foci, which led to chromosomal abnormalities, including loss of telomere maintenance; this was determined by detection of cH2Ax foci in interphase nuclei [57]. Following 4 weeks of Pb acetate exposure at 100 mg/BW, ROS and malondialdehyde levels, as indicators of oxidative stress, significantly increased in mouse livers and evoked DNA damage in mouse lymphocytes (2.43-fold and 2.48-fold for tail length and tail moment, resp.). In addition, lead acetate (10 mg/kg BW) increased the expression of p53 and Bax, leading to an imbalance in the Bax/Bcl-2 ratio; these proteins are associated with apoptosis [58]. Cr^6+^ induced bulky DNA adducts and oxidative DNA damage at 2′-deoxyadenosine and deoxyguanosine residues, contributing to mutations in p53 that are associated with lung cancer [59].

## 3. Discussion

Most cancers are the result of a combination of genetics and environment. Research shows that >90% of cancers are caused by external environmental factors that directly or indirectly affect DNA, leading to genetic defects. However, only approximately 5% of cancers are caused by genetic abnormalities [60, 61]. Molecular epidemiologic research has confirmed that environmental factors are the major causes of human cancer and the risk of developing these factors also depends on an individual's genetic and acquired susceptibility [62]. Environmental and lifestyle factors include diet, tobacco, infections, obesity, alcohol, radiation, stress, physical activity, and heavy metals pollution [60, 61]. With the development of industrialization and urbanization, heavy metals pollution has posed a serious threat to human health. According to the World Health Organization, approximately 130 million people may have been exposed to drinking water containing >10 g As/L [63]. Heavy metal exposure correlates with the incidence and mortality of gastric cancer. We have focused on four possible mechanisms by which heavy metals may induce gastric cancer (Table 2). The most relevant pathomechanism for heavy metal-driven carcinogenesis is barrier failure. The relationship between barrier failure and carcinogenesis is complex: barrier failure may trigger inflammation and carcinogenesis, but inflammation and carcinogenesis may also promote barrier failure, thus suggesting the existence of forward-amplifying loops [64]. Failure of the gastric mucosal barrier is considered a critical aspect of gastric cancer development. Once the gastric mucosa is damaged,* H. pylori* causes lesions in the stomach mucosa tissue more easily; this causes chronic inflammation and increases the incidence of gastric cancer. Recently, it has been show that Cd, Pb, and Cr, but not Hg or As, can cause stomach mucosal damage. However, Cd, Pb, Cr, As, and Hg directly or indirectly induce ROS generation, which causes gastric mucosal and DNA lesions, subsequently evoking a series of changes and ultimately leading to gastric cancer development. Heavy metals inhibit DNA damage repair and they are also involved in other mechanisms associated with gene abnormalities.

Studies have shown that the pH of drinking water (5.0, 7.0, and 8.0) does not seem to affect the lesions in the digestive tract caused by Cd poisoning [26]. Although blood Cd levels are significantly higher in GI cancer patients compared with healthy individuals, a multivariate regression model did not reveal a significant correlation between blood Cd concentrations and the risk of GI cancer [65]. A recent study has shown that downregulation of DENN/MADD domain-containing protein 2D may be a promising biomarker to predict early recurrence and progression in all types of gastric cancer [66]. miR-196a/-196b concentrations were significantly increased in gastric cancer tissues. Overexpression of miR-196a/-196b significantly correlated with gastric cancer cell migration and invasion [67]. Recently, it has been reported that miR-10b acts as a novel tumor suppressor and that it was significantly downregulated and partially silenced due to DNA hypermethylation in gastric cancer [68]. Circulating miR-18a in plasma is considered a stable and effective biomarker for detecting gastric cancer and monitoring tumor dynamics [69]. The metal mixture of As, Cd, and Pb leads to overexpression of miRs-10, miRs-154, miRs-375, and miRs-222, which are associated with cancer development. Upon exposure to this metal mixture, miRs-222, miRs-379, miRs-204, miRs-133, miRs-222, miRs-375, and miRs-154 are upregulated, affecting cellular processes, such as the inflammatory response, cell growth and proliferation, and cell death [30]. Therefore, it will be necessary to further study the development of heavy metal-associated cancer caused by miRNAs.

Human epidermal growth factor receptor 2 protein overexpression and gene amplification are important biomarkers for trastuzumab treatment in breast and gastric cancer patients. Regardless of gender, there is a statistically significant association between As soil levels and mortality in gastric cancer [7]. Cr levels in topsoil correlated with the mortality of upper GI tract cancer in women alone [7]. However, another study determined that soil As and nickel exposure had a significantly higher influence on colon, gastric, kidney, and liver cancer development in male patients than in female patients [8]. For the period 2000 to 2004, the gastric cancer incidence male : female ratio was approximately 2 : 1 worldwide [4]. The sex ratio difference in risk may be caused by increased long-term cigarette smoking and alcohol consumption in males [4]. Cigarette smoking and alcohol consumption are some of the main risk factors for gastric cancer [4]. Thus, it is necessary to evaluate the effects of heavy metal exposure in male and female patients.

Some metals such as Cr^6+^, As^3+^, and Cd^2+^ promote the invasion and metastasis of cancer cells, in part via the generation of ROS [33]. In addition, urokinase-type plasminogen activator receptor (uPAR) expression plays an important role in the invasion and metastasis of gastric cancers. One study found that Cd can provoke cell invasion in human gastric cancer cells due to the overexpression of uPAR via the ERK-1/2, NF-*κ*B, and AP-1 signaling pathways. Research has convincing evidence that Cd enhances gastric cancer cell invasion and transfer [70]. Therefore, given the other potential pathways by which heavy metals may induce gastric cancer, future studies should focus on the molecular mechanisms that heavy metals employ to trigger carcinogenicity.

## 4. Conclusions

**Figure 5:**
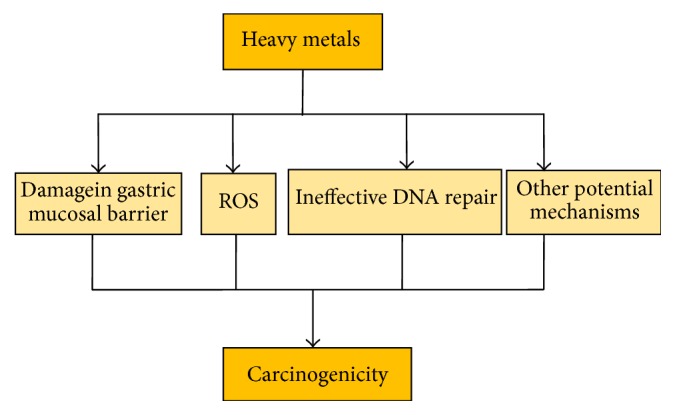
Mechanisms of heavy metal-induced carcinogenicity in the stomach [25].

There are four potential mechanisms by which heavy metals induce gastric cancer (Figure 5). Heavy metals disrupt the gastric mucosal barrier and lead to inflammation, tissue damage, and dysplastic lesions in the stomach glandular epithelium. Almost all heavy metals cause DNA lesions and enhance invasion and metastasis by cancer cells by generating ROS. Heavy metals also inhibit DNA damage repair or result in inefficient lesion repair and induce other gene abnormalities. In addition, heavy metals induce the expression of proinflammatory chemokine interleukin-8 (IL-8) and microRNAs in gastric cancer development.

To decrease the risk of gastric cancer, it is necessary to establish public health strategies, enhance the detection of environmental contaminants, and, in particular, control heavy metal pollution and the emission of pollutants. A recent study indicated that volatile organic compound biomarkers and breath analysis based on surface enhanced Raman scattering sensors can not only diagnose gastric cancer but also distinguish between early and advanced gastric cancer patients and healthy persons [71]. To develop a more robust health risk assessment, it will be necessary to study the relationship between heavy metal contamination and human health [72]. In addition, people should consume low molecular weight antioxidants such as vitamin C, vitamin E, flavonoids, and other antioxidants as these chelating metal ions can affect metal toxicity [73].

## Figures and Tables

**Table 1:** The relationship between heavy metal exposure and gastric cancer.

Heavy metal	Main findings	Reference
As	Gastric cancer increased by 8.2% in villages with As-contaminated drinking water.	[12]
As	The rates of GI cancers increased in an area in Turkey with high As contamination, compared with the average rate in Turkey.	[13]
Pb, As, Sb	Statistical analysis revealed a high correlation between gastric cancer and areas with mineral deposits of Pb, As, Sb.	[11]
Cr	Meta-analysis indicated that Cr^6+^ exposure increases the risk of gastric cancer (RR = 1.41, 95% CI 1.18, 1.69).	[3]
Cr	Cr topsoil concentrations correlated with mortality of upper GI tract and breast cancer among women.	[7]
Pb	Inorganic lead exposure is associated with gastric cancer (OR = 3.0, 95% Cl = 1.2–7.3; and OR = 2.0; 95% CI = 1.1–3.8, resp.).	[15]
As	Soil arsenic was significantly positively correlated with gastric cancer (*P* = 0.412); an increase of 1 ppm arsenic concentration in soil was associated with an 11.1% increase in the gastric cancer mortality rate.	[13]
As	As exposure was significantly associated with colon, gastric, kidney, lung, and nasopharyngeal cancer mortality rates.	[6]
Cd, Pb	Cd and Pb exposure increased the risk of mortality from all cancers, including stomach, esophageal, and lung cancers.	[7]
Pb	A significantly statistical association was observed between Pb topsoil levels and primary gastric cancer.	[17]
Cd	Gastric cancer patients had higher urine Cd concentrations in Tabriz, Northwest of Iran (OR = 1.70, 95% CI = 1.35–2.20).	[10]
Hg	The level of Hg in hair positively correlated with the transition of gastritis to superficial gastritis and atrophic gastritis or even cancer.	[14]

As, arsenic; Cd, cadmium; Cr, chromium; Hg, mercury; Pb, lead; Sm, antimony; CI, confidence interval; GI, gastrointestinal; RR, relative risk; OR, odds ratio.

**Table 2:** Main mechanism of gastric cancer induced by each kind of heavy metal.

Heavy metals	Main mechanism	Reference
Cd	(1) Disrupt the stomach mucosal barrier	[19–21, 23, 24, 26–30]
	(2) DNA damage via ROS	
	(3) Ineffective DNA repair	
	(4) Alter catalase activity	
	(5) Accelerate cancer development	

As	(1) Cell damage	[23, 24, 30–33]
	(2) Inhibit DNA repair	
	(3) Epigenome	
	(4) Enhanced cancer development by inducing overexpressing of miRNAs	

Hg	(1) DNA damage	[24, 28, 34–36]
	(2) Chromosomal aberration	
	(3) Induce immune dysfunction	

Pb	(1) DNA damage via ROS	[22–24, 28, 30, 37–39]
	(2) Ineffective DNA repair and inhibiting DNA repair with UItraviolet rays	
	(3) Promote tumorigenesis via Il-8	
	(4) Change catalase activity	
	(5) Enhanced cancer development by inducing overexpressing of miRNAs	

Cr	(1) DNA lesion	[23, 24, 32, 33, 40–42]
	(2) Gene abnormalities	
	(3) Promote cancer cell migration and invasion	
